# Supplementary material for: Hypoxia and loss of GCM1 expression prevents differentiation and contact inhibition in human trophoblast stem cells
Source: bioRxiv. 2024 Sep 10:2024.09.10.612343. Preprint. [Version 1] doi: 10.1101/2024.09.10.612343 (PMC11419009; doi:10.1101/2024.09.10.612343)
Supplement: Supplement 1 [file media-1.pdf]

Supplemental Tables

Table S1. FPKM, DEG (differentially expressed gene) analysis, z-score cluster analysis of hypoxia RNA-seq samples. Related to Figure 1.

Table S2. List of 100 specific cell type genes corresponding to FPKM of hypoxia samples. Related to Figure 2.

Table S3. FPKM for Control and GCM1-/- samples and DE-seq analysis. Related to Figure 2.

Table S4. Coordinates of ChIP-seq and ATAC-seq peak sets. Related to Figure 3.
