# Supplementary material for: Hypoxia and loss of GCM1 expression prevents differentiation and contact inhibition in human trophoblast stem cells
Source: bioRxiv. 2024 Sep 10:2024.09.10.612343. Preprint. [Version 1] doi: 10.1101/2024.09.10.612343 (PMC11419009; doi:10.1101/2024.09.10.612343)
Supplement: Supplement 6 [file NIHPP2024.09.10.612343v1-supplement-6.pdf]

bioRxiv preprint doi: https://doi.org/10.1101/2024.09.10.612343; this version posted September 10, 2024. The copyright holder for this preprint (which was not certified by peer review) is the author/funder, who has granted bioRxiv a license to display the preprint in perpetuity. It is made available under aCC-BY 4.0 International license.

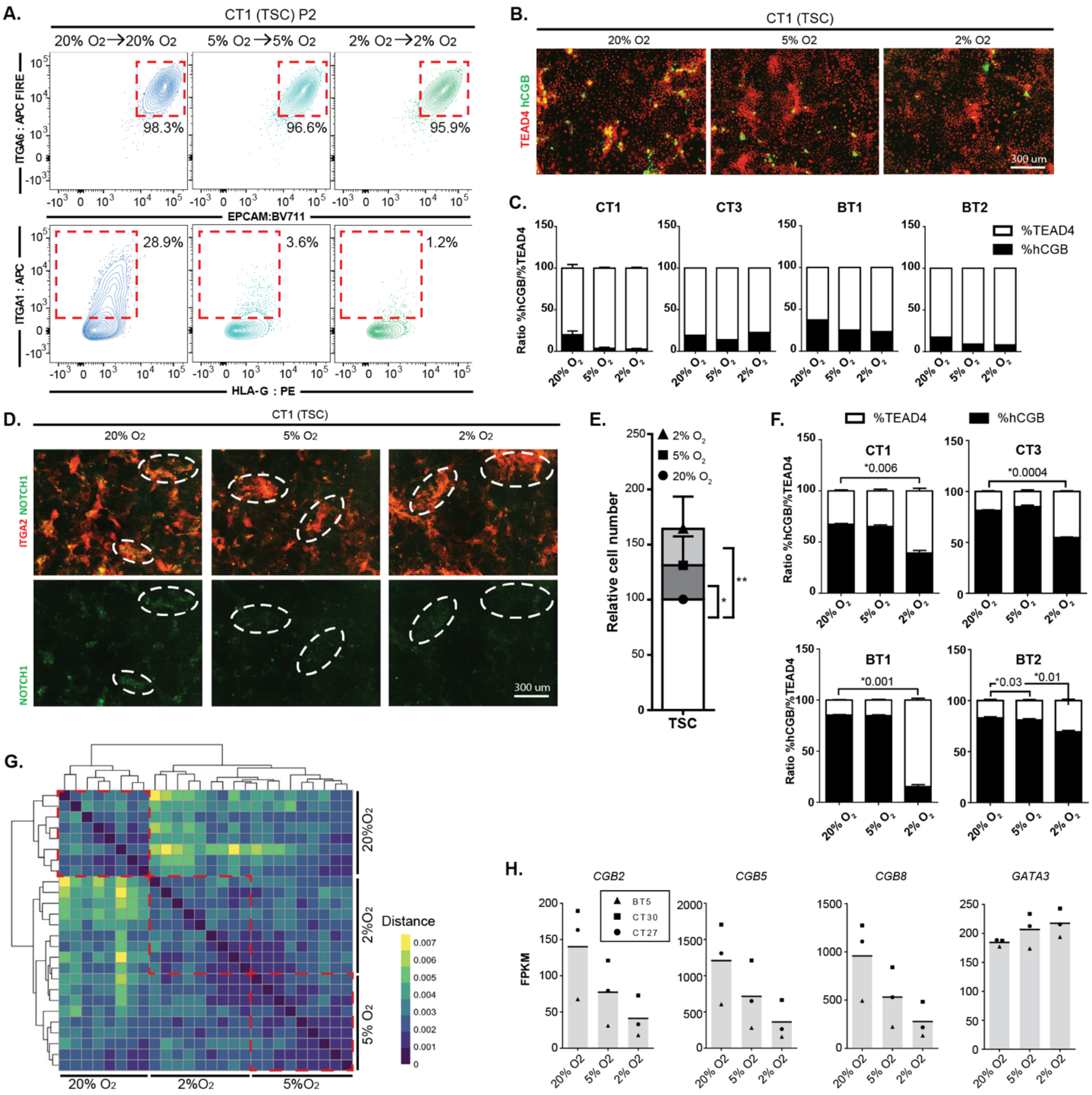

I.

Enriched transcription factor target sets Cluster1

351 genes (13.8%) from the input list are present in at least one TF target set.
The total number of genes present in at least one TF target set and identifiable by 'ensembl' IDs is 2373.

transcription factor	set size	candidates contained	p-value	q-value
HIF1A	82	30 (36.6%)	5.51e-07	0.000103
PTTG1	15	9 (60.0%)	6.74e-05	0.0063
MYCN	45	16 (35.6%)	0.000398	0.0248
MYC	99	25 (25.5%)	0.00316	0.129
VHL	20	8 (40.0%)	0.00523	0.129
HDGF	6	4 (66.7%)	0.00552	0.129
ARNTL	6	4 (66.7%)	0.00552	0.129
HIVEP2	6	4 (66.7%)	0.00552	0.129
CLOCK	13	6 (46.2%)	0.00686	0.143
TFDP1	10	5 (50.0%)	0.00914	0.171

J.

Enriched transcription factor target sets Cluster2

338 genes (11.5%) from the input list are present in at least one TF target set.
The total number of genes present in at least one TF target set and identifiable by 'ensembl' IDs is 2373.

transcription factor	set size	candidates contained	p-value	q-value
GCM1	4	4 (100.0%)	0.000405	0.0595
ATF4	35	13 (37.1%)	0.000601	0.0595
KLF6	20	8 (40.0%)	0.00411	0.217
HIC1	9	5 (55.6%)	0.00439	0.217
DDIT3	18	7 (38.9%)	0.00866	0.269
PPARG	66	17 (25.8%)	0.00878	0.269

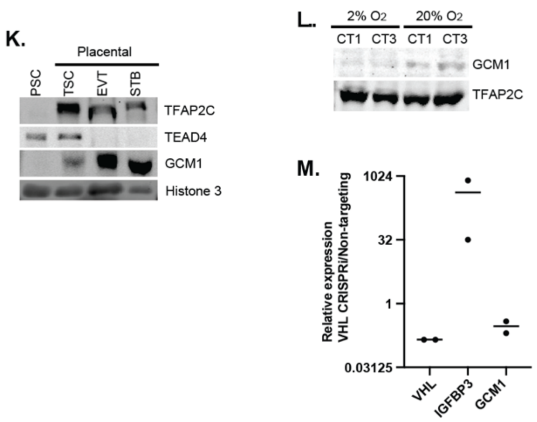

bioRxiv preprint doi: https://doi.org/10.1101/2024.09.10.612343; this version posted September 10, 2024. The copyright holder for this preprint (which was not certified by peer review) is the author/funder, who has granted bioRxiv a license to display the preprint in perpetuity. It is made available under aCC-BY 4.0 International license.

Figure S1. Reduced and impaired hTSC differentiation in hypoxic conditions. A. Trophoblast stem cells shown in Figure. 1A were passaged and cultured for additional 72hrs in varying levels of oxygen (20%, 5%, 2% O_2). Continued low oxygen tension causes further reduction of $ITGA1^+$ cell population. **B.** Spontaneous differentiation of hTSC to STB in as indicated by loss of TEAD4 and gain of hCGB. Note trend toward higher hCGB staining at 20% O_2. **C.** Quantification of spontaneous hCGB expression across multiple cell lines at oxygen concentration indicated (4 cell lines, n=4 for each cell line). **D.** hTSC in the varying oxygen conditions were grown to over maximum confluency. At regions where overgrowth causes increase cell-to-cell contact and cell pile-up, spontaneous nuclear NOTCH1 signal is observed. In the low oxygen cultures, NOTCH1 expression is not detected. **E.** Relative numbers of TEAD4+ cells per unit area (4 cell lines, n=4 for each cell line). **F.** Quantification of spontaneous hCGB expression across multiple cell lines at oxygen concentration indicated (4 cell lines, n=4 for each cell line). **G.** Correlation matrix showing sample clustering of RNA-seq data from hTSCs in culture conditions indicated (3 cell lines, n=3 for each line in each condition). **H.** Bar graphs showing FPKM of specific genes of interest (n=3 replicates, except BT2 at 20% O_2 n=2). **I., J.** ConsensusPathwayDB analysis of Cluster 1 and Cluster 2 from Figure 1H, identifying TF targets with significance from each cluster. **K.** Western blot comparing pluripotent stem cell (PSC) that don't express placental markers, with hTSC and differentiated EVT and STB to highlight specific expression patterns. **L.** Western blot for GCM1 in 2 cell lines grown in 2% and 20% O_2. TFAP2C is the loading control. **M.** Ratio of expression for genes indicated in hTSCs subjected to CRISPRi-targeted degradation of VHL relative to control hTSC. Note reduced expression of GCM1, while known hypoxia target IGFBP3 is dramatically upregulated.

bioRxiv preprint doi: https://doi.org/10.1101/2024.09.10.612343; this version posted September 10, 2024. The copyright holder for this preprint (which was not certified by peer review) is the author/funder, who has granted bioRxiv a license to display the preprint in perpetuity. It is made available under aCC-BY 4.0 International license.

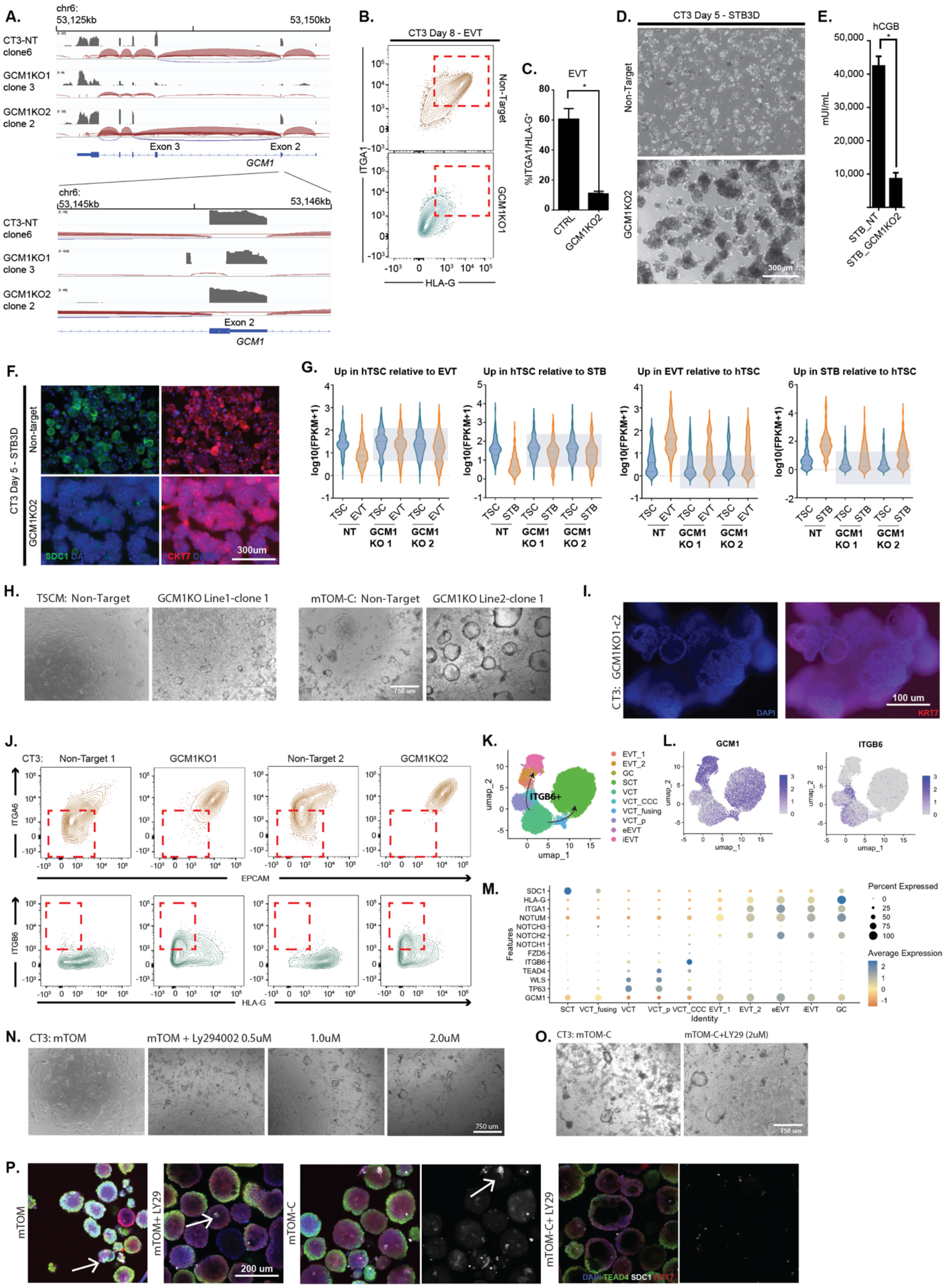

bioRxiv preprint doi: https://doi.org/10.1101/2024.09.10.612343; this version posted September 10, 2024. The copyright holder for this preprint (which was not certified by peer review) is the author/funder, who has granted bioRxiv a license to display the preprint in perpetuity. It is made available under aCC-BY 4.0 International license.

Figure S2. Impaired differentiation upon genetic or chemical reduction in GCM1 level. A. Sashimi plot across the genomic region of *GCM1*. Representative clones were chosen. Normal splicing is observed from non-target line. *GCM1*$^{-/-}$ Line1 (from right to left) show a deletion at the distal tip of exon 2 but an alternative slice site forming just after. *GCM1*$^{-/-}$ Line 2 shows the complete skipping of exon 3. **B.** Flow cytometric analysis from EVT differentiation of *GCM1*$^{-/-}$ Line 2 and NT hTSC. NT cells differentiation produce ITGA1^{hi}/HLA-G^{hi} cells whereas *GCM1*$^{-/-}$ TSC do not. **C.** Bar graph showing formation of ITGA1^{hi}/HLA-G^{hi} population from control and *GCM1*$^{-/-}$ Line 2 hTSC (n=3 replicates). **D.** STB3D formation of NT and *GCM1*$^{-/-}$ Line 2 hTSC. Control hTSCs form a fluid-filled syncytium while *GCM1*$^{-/-}$ form a cluster of cells. **E.** hCGB ELISA was perform using supernatant from *GCM1*$^{-/-}$ and control hTSC (n=3 replicates). **F.** Control and *GCM1*$^{-/-}$ Line 2 STB3D stained for the STB-marker SDC1 and the pan-placental marker CKT7. Note absence of SDC1 in *GCM1*$^{-/-}$. **G.** Violin plot showing expression of genes specific to hTSC, EVT or STB in cell types indicated. Differentiated *GCM1*$^{-/-}$ cells fail to express differentiation markers and retain expression of TSC markers instead. **H.** *GCM1*$^{-/-}$-hTSC were grown in mTOM with and without CHIR99021. Dome-like projections appeared in regions of high cell density. **I.** Immunofluorescent staining of *GCM1-/-* trophoblast organoids. **J.** Flow cytometry of NT and *GCM1*$^{-/-}$ -3D-TSC differentiated to EVT. *GCM1*$^{-/-}$ hTSCs fail to upregulate the EVT marker HLA-G but do upregulate the cell-column marker, ITGB6. **K.-M** Reanalysis of Arut. *et al.* 2022 single RNA-seq profiling the several subtypes found in early villus of the placenta. **K.** Cell types in placenta, with path of differentiation indicated. (GC=Giant Cell, VCT = villous CTB, VCT_p=proliferating CTB, VCT_CCC = cell column cytotrophoblast eEVT=endovascular EVT, iEVT= interstitial EVT). **L.** Expression of GCM1 and ITGB6 in cells shown in (**K**). **M.** Expression of genes indicated in cell types indicated. Note that ITGB6 is associated with cell column cytotrophoblast. **N.** hTSC cultured in mTOM media with varying quantities of LY294002. **O.** hTSC cultured in mTOM-C with or without LY294002 2μM. **P.** day14 TB-ORG grown in mTOM or mTOM-C with or without LY294002 2μM treatment and IF stained for DAPI, TEAD4, SDC1, and KRT7. Arrows mark areas of SDC1expression.

bioRxiv preprint doi: https://doi.org/10.1101/2024.09.10.612343; this version posted September 10, 2024. The copyright holder for this preprint (which was not certified by peer review) is the author/funder, who has granted bioRxiv a license to display the preprint in perpetuity. It is made available under aCC-BY 4.0 International license.

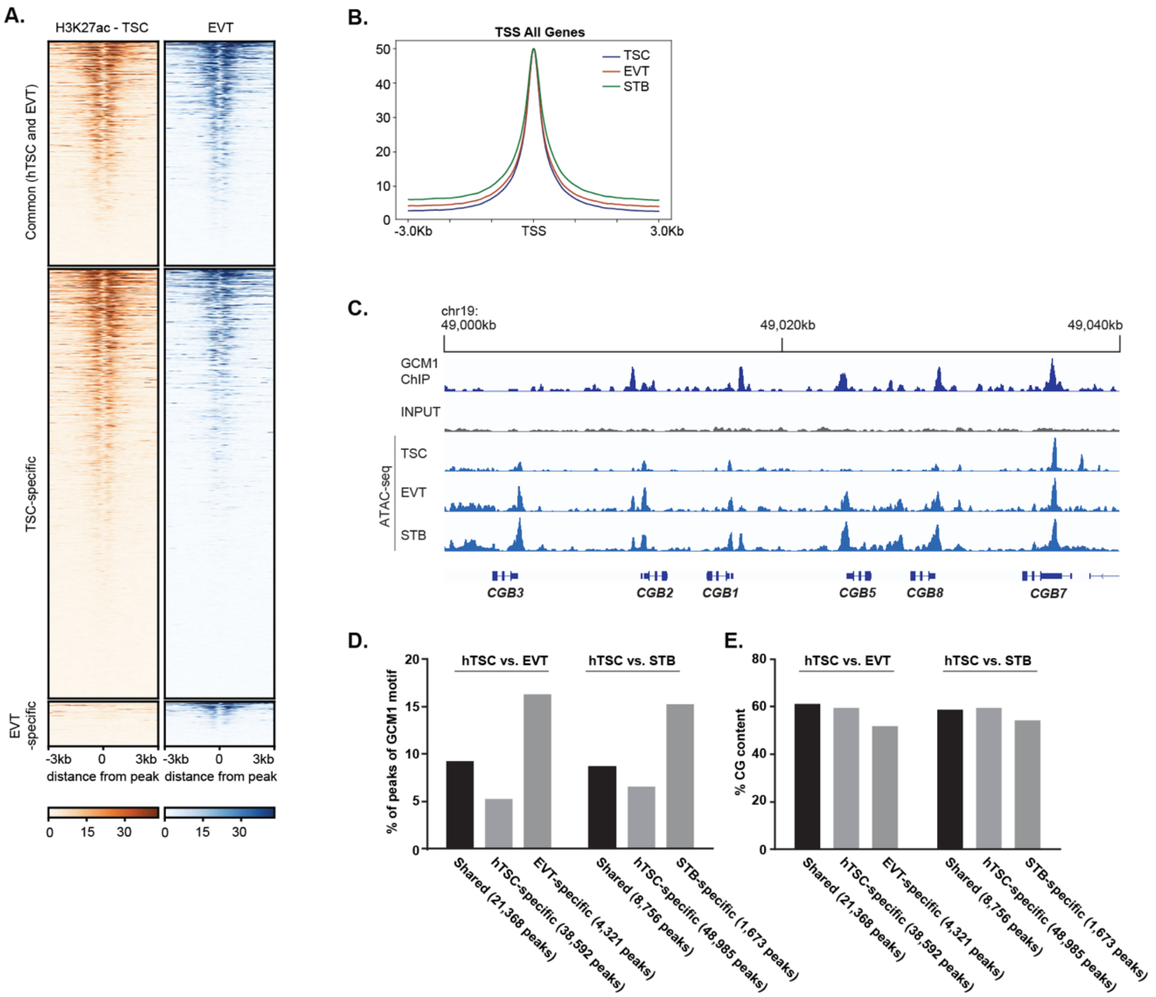

Figure S3. GCM1 positively regulates differentiation-associated genes. A. Heatmap of H3K27Ac enrichment over common, hTSC-specific, and EVT-specific ATAC-seq peaks in hTSC and EVT. Note correspondence of H3K27Ac enrichment with ATAC enrichment in each set. **B.** Metaplot of ATAC-seq data from TSC, EVT and STB over all gene TSS after normalization. **C.** GCM1 ChIP-seq and ATAC-seq data plotted over the *CGB* locus. **D.** Percentage of peaks in each category containing GCM motifs. **E.** GC content of ATAC-seq peaks in each category. GCM1 has a GC-rich motif, but the higher frequency of GCM1 sites observed in (**D**) cannot be explained by difference in underlying GC richness.
